# Pancreatic Cancer: Feasibility and Outcome After Radiochemotherapy with High Dose External Radiotherapy for Non-resected and R1 Resected Patients

**DOI:** 10.7759/cureus.2713

**Published:** 2018-05-31

**Authors:** David C Lauffer, Peter A Kuhn, Marc Kueng, Sandrine U Thalmann, Géraldine Risse, Pierre-Alain Tercier, Bernhard Egger, Abdelkarim S Allal

**Affiliations:** 1 Department of Radiation Oncology, Hospital of Fribourg, Fribourg, CHE; 2 Department of Medical Oncology, Hospital of Fribourg, Fribourg, CHE; 3 Department of General Surgery, Hospital of Fribourg, Fribourg, CHE

**Keywords:** pancreatic adenocarcinoma, high dose, radiotherapy, survival, toxicities

## Abstract

Background

Non-resected locally advanced and microscopic positive-margin resected (R1) pancreatic adenocarcinoma are associated with a dismal prognosis. The combination of high dose radiotherapy and concomitant chemotherapy is among the strategies that are used to improve the outcome. The aims of this study were to evaluate the acute and late toxicities and patients' outcome in a retrospective study from a single center.

Material and methods

From 2009 to 2015, 24 patients, with non-resected locally advanced or R1 resected pancreatic adenocarcinoma, have been treated with concomitant radiochemotherapy, with a median dose of 60 Gy and gemcitabine (50 mg/m^2^ administered bi-weekly). The acute and late toxicities were evaluated during and after the treatment.

Results

The actuarial overall survival rates were 39% at 24 months and 8.6% at 36 months. The disease-free survival rates were 32.5% at 24 months and 12.2% at 36 months. Acute toxicities were mainly grade 1 (G1) to grade 2 (G2) except for one patient who presented with severe digestive bleeding potentially linked to the treatment. Late toxicities consisted mainly of G1 digestive toxicities.

Conclusion

This study confirms the feasibility of high dose radiotherapy combined with gemcitabine-based chemotherapy in patients with locally advanced pancreatic adenocarcinoma. While the outcome remains unsatisfactory, some patients seem to have benefited from this aggressive therapy, which merits to be investigated further.

## Introduction

Pancreatic carcinoma is a rare cancer compared to breast, prostate, and lung cancers. Cancers account for 7% of all deaths in the US, with pancreatic cancer as the fourth most common cause after lung (26%), colorectal (8.5%), and prostate (8%) cancers for men, and breast (14%) cancer for women [1-3], making this cancer one of the most deadly.

The anatomic position of the pancreatic gland in the retroperitoneal space makes it close to many important organs such as stomach, duodenum, and main arteries and veins. Furthermore, pancreatic cancer is characterized by the propensity to progress silently locally and to spread to regional lymphatics. Because of these reasons, at diagnostic, a large percentage of patients (80% to 85%) present an extension to adjacent organs, lymph nodes, fat, soft tissue [4,5], or distant metastases. This makes complete surgical resection difficult, if not impossible [6], with an estimated complete surgical resection of around 10 to 12% [7].

The only treatment that potentially offers a definitive cure with a long-term survival is a surgical resection with negative margin (R0). In this setting, the reported five-year overall survival rate is about 20% [8-10]. Either the tumorʼs extension or the poor performance status (PS) of those patients make the majority of them not operable, and among them only a small part will have a negative surgical margin [11]. A substantial number of patients will have microscopic positive-margin (R1) making their prognosis close to those with unresectable tumors with a five-year overall survival < 5% [12].

For patients with locally advanced, unresectable non-metastatic disease, multimodal treatments, including radiotherapy and chemotherapy, remain a challenge as for R1 resected patients [13-15]. Several studies, including questionable ones, have tried to establish the role of multimodal approaches, but due to some controversial and conflicting results, the definitive role of these modalities is not yet well established. These treatments have improved only slightly the median survival time to 8-14 months [16-21] despite the improved control of micro-metastasis by the modern systemic therapy regimens [22]. Here we report the results of a combined high dose radiotherapy (RT) with concurrent gemcitabine for locally advanced or R1 resected pancreatic cancers.

## Materials and methods

Between March 2009 and August 2015, 24 patients (11 females and 13 males) from a single institution (Department of Radiation Oncology at the Hospital of Fribourg, Switzerland) were enrolled in a retrospective study. Eighteen patients had non-resected locally advanced and six had R1 resected pancreatic carcinoma. Histologic confirmation of pancreatic adenocarcinoma was established in all patients. The tumors were considered unresectable either during standard diagnostic workup or after exploratory laparotomy. Four patients presented with cT2-3, 14 with cT4, and six with pT3-4.

Before radiotherapy, all patients underwent a general physical examination. A spiral computed tomography scanning of the upper abdomen in treatment position was performed in all patients. Target delineation was based on data collected and merged from the different imagery procedures and concerned mainly the macroscopic/microscopic areas while elective regional nodes were considered if organs at risk could be well preserved. The mean gross tumour volume (GTV) to clinical target volume (CTV) margin was 5.2 mm (range: 5-6 mm). The mean CTV to planning target volume 1 (PTV1) margin (tumor + lymph nodes) was 7.4 mm (range: 5-10 mm). The mean CTV to PTV2 margin (tumor/bed) was 6.3 mm (range: 5-10 mm). All patients received a dose of 59 Gy or more (median dose 60 Gy) except one patient who received 56 Gy. The dose per fraction was mainly between 1.8 and 2 Gy. All patients were treated in supine position; four patients were treated with volumetric modulated arc therapy (VMAT) (TrueBeam, Varian Medical Systems, Inc., CA, USA), 10 patients were treated with intensity modulated radiation therapy (IMRT, TomoTherapy), while the rest were treated with three-dimensional techniques using multiple fields with customized blocks. Special attention was paid to the maximal doses allowed to organs at risk (Table 1). Chemotherapy was based on gemcitabine (50 mg/m^2^ administered bi-weekly).

**Table 1:** Dose to organs at risk (Gy) *LN*: lymph nodes. *PTV*: planning target volume.

Organs	Mean	Minimum	Maximum
Liver	11.45	0.53	57.80
Stomach	17.43	1.17	54.35
Right kidney	8.67	1.57	32.20
Left kidney	6.47	1.40	28.14
PTV1 (Tumor + LN)	55.62	42.54	61.94
PTV2 (Tumor)	59.82	53.99	62.19

A physical examination and an evaluation of acute toxicity were performed on a weekly basis. After the treatment, patients were followed on a regular basis. The acute and late toxicities were evaluated according to common terminology criteria for adverse events (CTCAE) v3.0 during and after treatment. Overall and disease-free survival rates were calculated from the date of diagnosis. The median follow-up was 8.0 months (range 1-42 months). All 24 patients were evaluated for toxicity and survival.

## Results

Acute toxicities

The majority of patients experienced grade 1 (G1) to grade 2 (G2) acute toxicity except for one patient who presented with severe digestive bleeding, potentially treatment-related. Grade 1 toxicities were observed in 58.3% of patients (14 out of 24) and grade 2 in 29.2% (seven out of 24), and consisted of fatigue, nausea, emesis, and weight loss. Grade 3 toxicities were experienced by 12.5% of patients (three out of 24) and consisted mainly of asthenia occurring at the end of the treatment.

Late toxicities

Late toxicities consisted mainly of G1 digestive toxicities. Grade 1 toxicities were observed in 83.3% (20 of 24) and G2 toxicities in 4.2% (1 of 24). Two patients died within three months after the completion of radiotherapy but without known toxicities. Three patients presented with digestive bleeding and two patients presented with an ileus. All these five later patients were known to have a loco-regional disease progression.

Oncologic results

The actuarial overall survival rates were 70% at 12 months, 39% at 24 months, and 8.6% at 36 months. The disease-free survival (DFS) rates were 48.8% at 12 months, 32.5% at 24 months, and 12.2% at 36 months. According to the tumor status, the DFS rates were of 83.3% and 41.7% at 12 and 36 months for R1 resected patients and 70% and 10% for non-operated patients. For all patients, the loco-regional control (LRC) rates were of 67.3%, 56.1%, and 21.0% at 12, 24, and 36 months, respectively. More than 70% of the patients were deceased at the time of this analysis. Although the causes of death were sometimes difficult to determine (death at home, no autopsy), we noted that most of deaths were due to loco-regional or distant progression. Local progression consisted of stomach and duodenum invasion while distant progression consisted mainly of metastases to the liver, lungs, or the peritoneum.

## Discussion

Treatment of patients with unresected or R1 resected pancreatic cancer remains a challenge. Without any treatment, the prognosis is very poor. The treatment may consist of a combined radiochemotherapy as described in the present study. Due to the retrospective nature of our study we are aware of the limitations and the caution that we should have in interpreting the results. Nevertheless, in line with some published data, the current study confirms the feasibility of high dose RT combined with gemcitabine-based chemotherapy with acceptable acute and late toxicities.

There is relatively less information on the behaviour of R1 resected patients treated with adjuvant radiotherapy and most of them come from sub-group analysis. Even though the number of patients is quite limited, the results showed in this study for R1 resected patients were slightly better than those of non-operated patients.

At three years, Blackstock et al. [16] reported an overall survival (OS) rate of 5% for patients with unresectable tumors treated by radiotherapy (50.4 Gy) and gemcitabine. Our results compare favorably with an OS of 8.6% at three years; however, our study includes 25% of R1 resected patients. On the other hand, the OS of R1 patients in our study is at least as good as the one reported in the radiation therapy oncology group (RTOG) 9704 study [23].

Willett et al. [24], observed a decreased death risk with adjuvant chemoradiotherapy in R0 resected patients compared to R1 patients with 29% vs 18% five-year OS. No patients with R1 margins survive beyond 41 months. This confirms the association of R1 margins with a worse outcome and also the relevance of considering adjuvant local and systemic treatment for these patients.

Rwigema et al. [25] reported that patients with R1 resected tumors benefit from adjuvant stereotactic external beam RT (SBRT), with a two-year OS rate of 61.4%. This was higher than the two-year OS rate of 39% for similar patients receiving adjuvant gemcitabine alone observed in the study of Oettle et al. [8]. A recent study by Chang et al. [26], showed results suggesting that patients with close surgical margins may benefit from more aggressive therapeutic approaches such as SBRT that target loco-regional disease.

Treatment of locally unresectable pancreatic cancer remains highly challenging. Among the attempts to improve the outcome, multimodal approaches such as radiotherapy plus chemotherapy have been regularly explored. Because of the risk toxicity particularly to gastrointestinal tracts, RT doses have been historically limited, delivered at best with a maximum dose of 50 Gy or with treatment interruption halfway through. This made the interpretation of the results quite difficult [9,27,28]. An early study of the Gastrointestinal Tumor Study Group (GITSG) attempted a dose escalation and compared 40 Gy with 60 Gy, combined with chemotherapy. No improvement in OS or in local control was seen in the higher dose arm. Here again the doses were increased but in courses of 20 Gy with two-week breaks. These RT schemes are known to be less effective than the continuous ones and may contribute to a loss in the treatment efficacy [29].

On the other hand Krishnan et al. [14] did an interesting study at the MD Anderson Cancer Center, by comparing the outcomes of patients with locally advanced pancreatic cancer (LAPC) who were treated with curative intent either by dose-escalated IMRT or by standard fractionation radiation therapy regimens to a median dose of 50.4 Gy. Concurrent capecitabine was the main chemotherapy regimen used. They noticed that patients who received a dose with a biologically effective dose (BED) > 70 Gy had a superior OS and an improved recurrence-free survival (RFS). Interestingly, no additional toxicity in the high-dose group was observed. Higher dose (BED) was the only predictor of improved OS on multivariate analysis. They concluded that radiation dose escalation during consolidative chemoradiation therapy after induction chemotherapy for LAPC patients improves OS and loco-regional RFS. They also suggest that the choice of the concurrent chemotherapy regimen may have an impact on the tolerability; capecitabine being possibly better tolerated than gemcitabine, cisplatin/5-FU, or 5-FU/mitomycin C.

Based on the National Cancer Data Base (NCDB), Zhong et al. [30] compared the clinical outcomes of patients treated with either conventional fractionated RT (CFRT) or SBRT for locally advanced, non-metastatic pancreatic adenocarcinoma. The two cohorts were not exactly identical—the patients in the SBRT cohort were older, had a lower rate of chemotherapy utilization, and a lower proportion of T4 tumors while the patients in the CFRT cohort had a larger proportion of positive-node status. The RT dose in the CFRT group was of 50.4 Gy, while in the SBRT arm it was of 40 Gy (median dose per fraction of 8 Gy). The two-year OS rate was significantly better in the SBRT group compared to CFRT group, with 20.3% versus 16.3%. They didn’t find any subgroup that appeared to significantly benefit from CFRT versus SBRT. However, in order to validate those first results a confirmation from randomized trials is warranted.

## Conclusions

The present study confirms the feasibility of high dose RT combined with gemcitabine-based chemotherapy in patients with locally advanced pancreatic adenocarcinoma. While the outcome remains unsatisfactory in this disease, some patients seem to have benefited from this aggressive therapy that merits to be investigated further. The other emerging RT strategy, namely SBRT, showed encouraging results and merits future investigations too. The high rates of distant metastasis stress the need for more efficient systemic therapies.
