# Correction: Pancreatic Cancer: Feasibility and Outcome After Radiochemotherapy with High Dose External Radiotherapy for Non-resected and R1 Resected Patients

**DOI:** 10.7759/cureus.c14

**Published:** 2018-08-08

**Authors:** David C Lauffer, Peter A Kuhn, Marc Kueng, Sandrine U Thalmann, Géraldine Risse, Pierre-Alain Tercier, Bernhard Egger, Abdelkarim S Allal

**Affiliations:** 1 Department of Radiation Oncology, Hospital of Fribourg, Fribourg, CHE; 2 Department of Medical Oncology, Hospital of Fribourg, Fribourg, CHE; 3 Department of General Surgery, Hospital of Fribourg, Fribourg, CHE

Primary and first author David C. Lauffer's affiliation (Hospital of Fribourg) was incorrectly listed as located in Bern, CHE when in fact it is located in Fribourg, CHE. The author's affiliation has been updated to reflect this change.

